# Supplementary material for: Context-dependent coordination of movement in Tribolium castaneum larvae
Source: J Exp Biol. 2025 Apr 10;228(7):jeb250015. doi: 10.1242/jeb.250015 (PMC12045640; doi:10.1242/jeb.250015)
Supplement: Supplementary information [file jexbio-228-250015-s1.pdf]

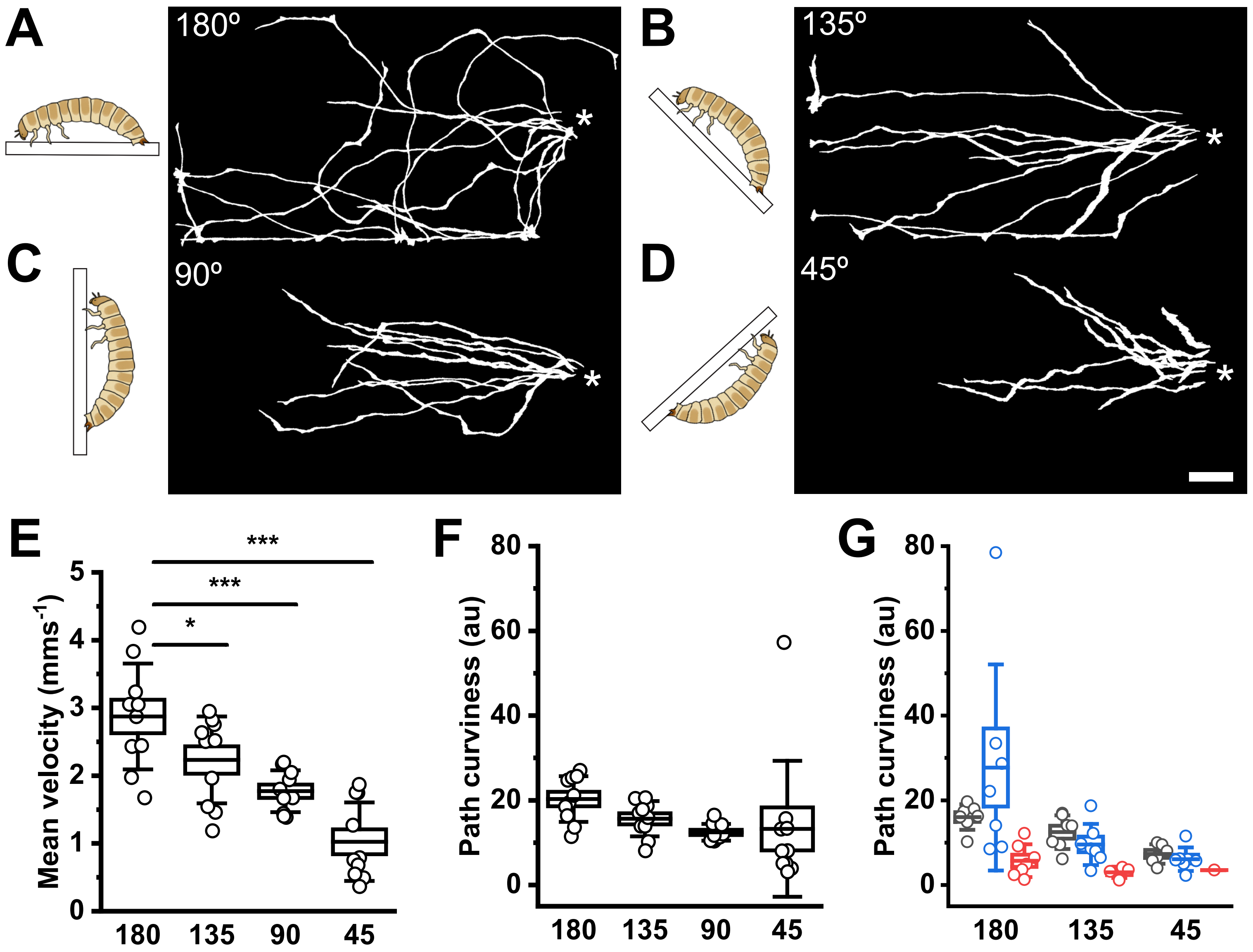

Fig. S1. Increasing incline becomes progressive locomotor challenge for *Tribolium* larvae. (A-D) Left: diagrams of larval orientations relative to angled platforms (white rectangles). Right: walk paths of larvae on respective platforms. Note, 45° is an overhang. Asterisks indicate starting position. (E) Mean instantaneous velocity across inclinations. Boxes = mean ±SE, whiskers = SD; *** p<.001, * p<.05, One-way ANOVA, n = 10. (F) Path curviness across inclinations calculated via (total distance / displacement) * max distance from start for preliminary control trials in (A-E), n = 10. (G) Path curviness for experimental trials in Fig. 5A,B across inclinations and surgical manipulations, n = 7.

Journal of Experimental Biology • Supplementary information

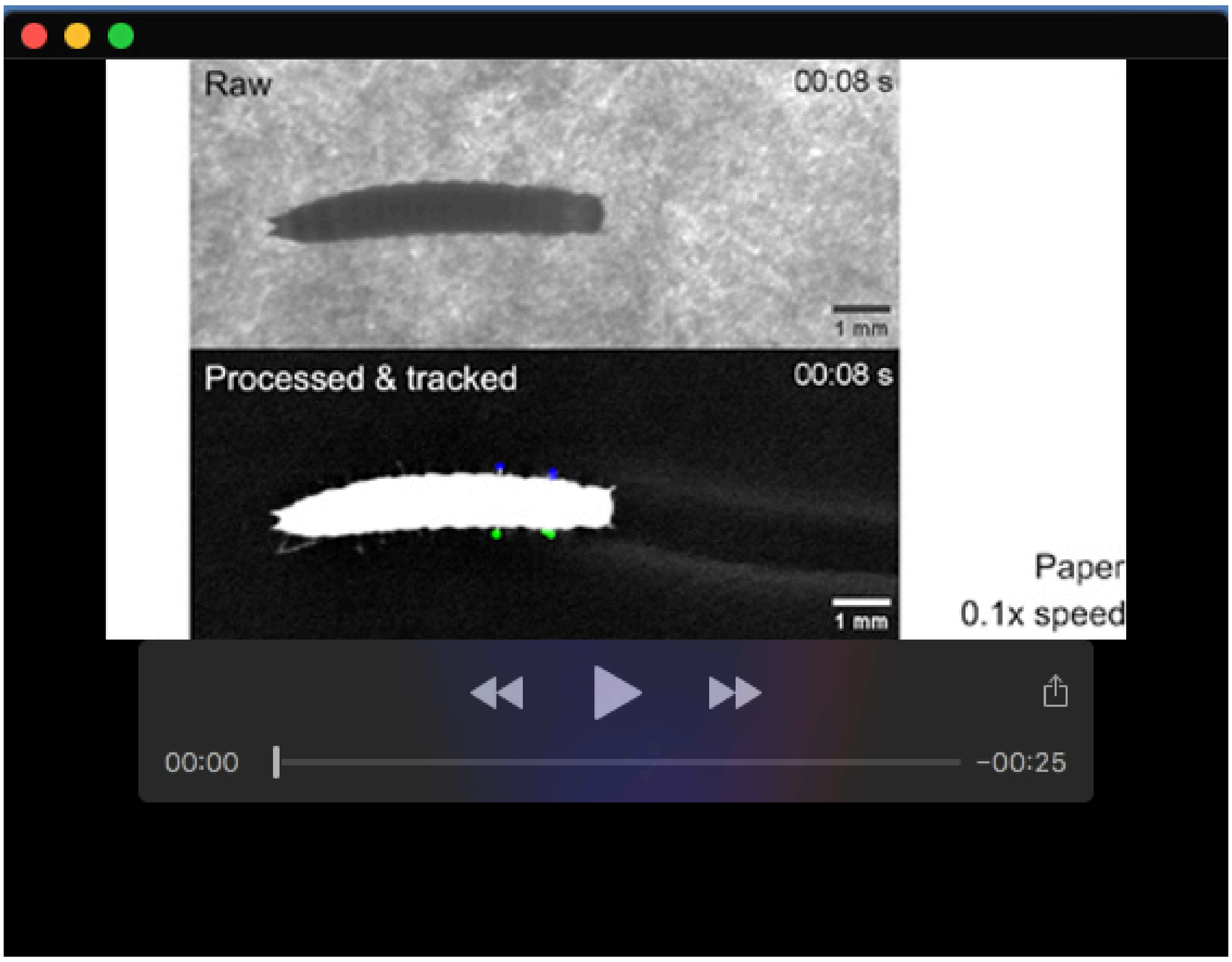

Movie 1. Natural larval locomotion on different substrates (white paper and 1% agarose).

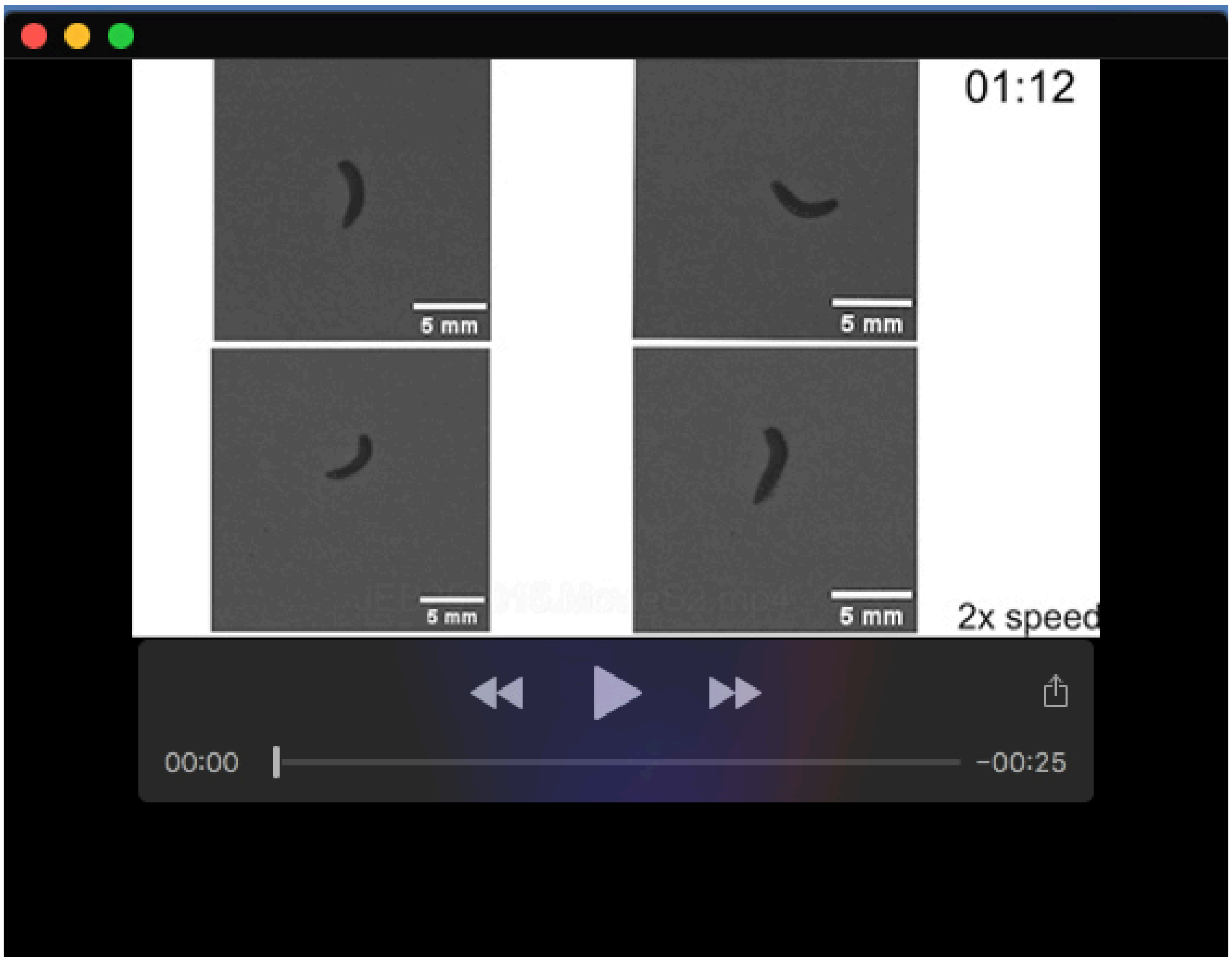

Movie 2. Backtrack and redirect behaviors in different larvae locomoting on paper.

Journal of Experimental Biology • Supplementary information

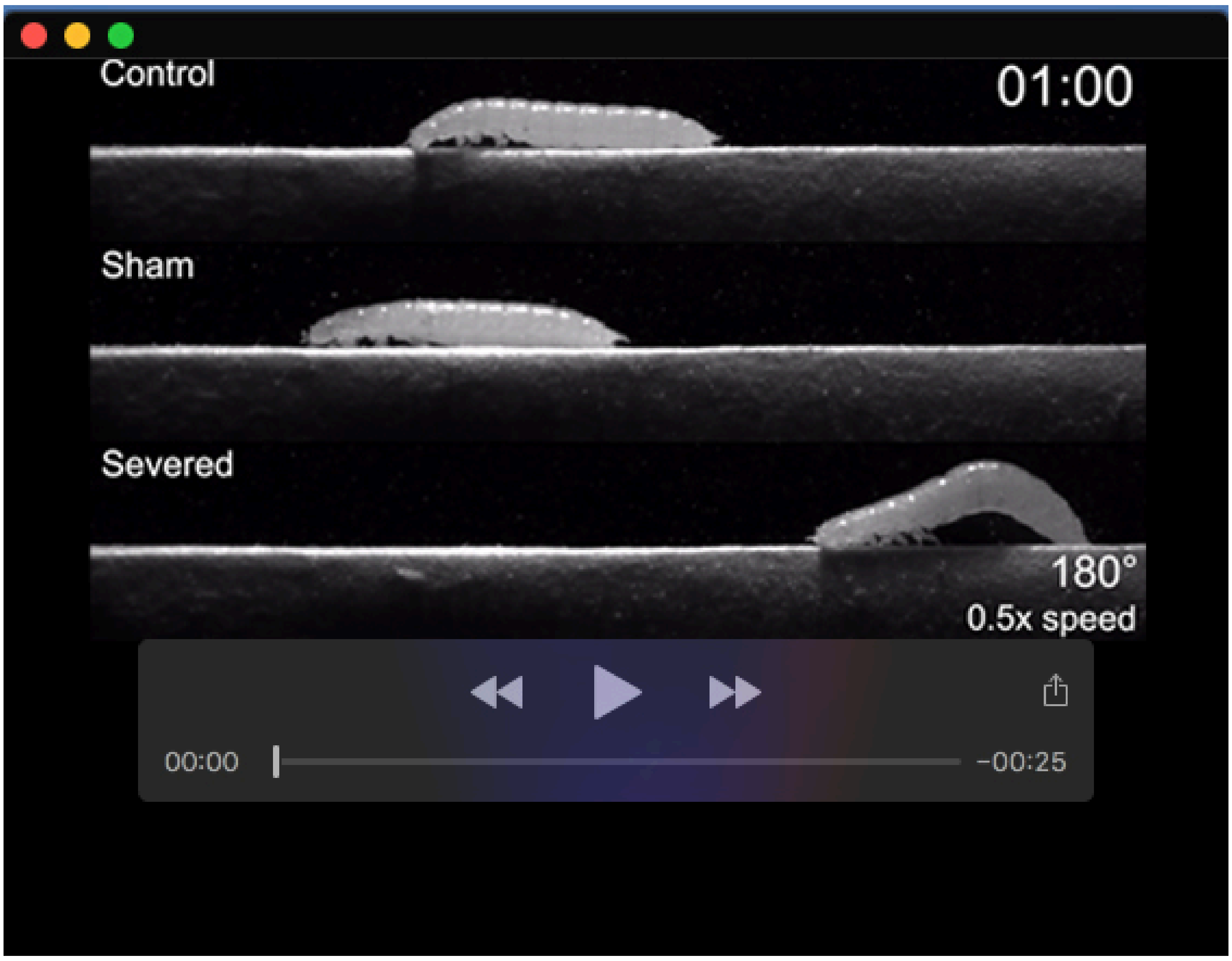

Movie 3. Control, sham, and severed larvae crawling on flat platform and climbing overhang platform. On the overhang, control and sham display pygopod planting while severed do not.
